# Clinical Mimickers of Mpox: A Case Series from a Tropical Referral Center

**DOI:** 10.3390/pathogens15101014

**Published:** 2026-09-25

**Authors:** Silvia Hees de Carvalho, Adriana de Souza Andrade, Janaina de Moura Freire, Marco Antônio Campos, Erna Geessien Kroon

**Affiliations:** 1 Serviço de Doenças Infecciosas e Parasitárias, Hospital Eduardo de Menezes, Fundação Hospitalar do Estado de Minas Gerais, Belo Horizonte 30622-020, MG, Brazil; 2 Instituto Nacional de Ciência e Tecnologia em Poxvírus, Belo Horizonte 31270-201, MG, Brazilmarco.campos@fiocruz.br (M.A.C.); 3 Fundação Oswaldo Cruz, Instituto René Rachou, Belo Horizonte 30190-009, MG, Brazil; 4 Laboratório de Vírus, Departamento de Microbiologia, Universidade Federal de Minas Gerais, Belo Horizonte 31270-201, MG, Brazil

**Keywords:** mpox, differential diagnosis, skin diseases, tropical medicine, lesions suspected of being mpox

## Abstract

Mpox is a zoonotic disease caused by the monkeypox virus, a double-stranded DNA virus belonging to the genus *Orthopoxvirus*. The World Health Organization (WHO) declared mpox a Public Health Emergency of International Concern (PHEIC) in July 2022 amid the global clade IIb outbreak and again in August 2024 following an increase in cases in the Democratic Republic of the Congo associated with clades Ia and Ib. Despite increased awareness, diagnosing mpox remains challenging because its clinical manifestations often overlap with those of other infectious and non-infectious conditions. This diagnostic challenge occurs across geographic settings but may be further complicated in tropical regions by the circulation of locally prevalent infections, including arboviral diseases such as dengue and chikungunya. Here, we describe a series of nine patients initially evaluated for suspected mpox, in whom alternative diagnoses were subsequently established.

## 1. Introduction

Mpox is a zoonotic disease caused by the monkeypox virus (MPV) [1], a member of the species *Orthopoxvirus monkeypox*, genus *Orthopoxvirus*, family *Poxviridae* [2]. Although historically considered a rare and self-limiting infection primarily endemic to Central and West Africa, the global epidemiological profile of mpox has changed significantly in recent years. MPV is classified into two major clades, historically associated with Central Africa (clade I) and West Africa (clade II), each divided into two subclades: Ia and Ib within clade I, and IIa and IIb within clade II. These genetically distinct subclades exhibit different epidemiological and clinical patterns [3,4].

Differences in genetic, epidemiological, and clinical characteristics were identified. Clade I viruses are associated with more severe disease and higher case-fatality rates than Clade II viruses. In contrast, Clade IIa typically causes milder disease with lower mortality. Clade IIb, including lineage B.1, was responsible for the global multi-country outbreak that began in 2022 because of its sustained human-to-human transmission [5].

These widespread international outbreaks of mpox prompted the World Health Organization (WHO) to declare the disease a Public Health Emergency of International Concern (PHEIC). More recently, Clade I was detected in several countries outside Africa [6]. In recent outbreaks, efficient human-to-human transmission was identified, including transmission through close physical contact and sexual contact, particularly among men who have sex with men (MSM). Transmission occurs through direct contact with infected lesions, bodily fluids, contaminated objects, and respiratory droplets. Although mpox is not traditionally considered a sexually transmitted disease, sexual contact has become an important route of transmission in recent outbreaks [7].

In Brazil, more than 10,000 confirmed or probable mpox cases were reported in 2022, followed by 853 cases in 2023. Mpox transmission persisted thereafter, with 2015 confirmed or probable cases reported nationwide between epidemiological week 1 of 2024 and epidemiological week 1 of 2025. In Minas Gerais, 406 suspected cases were reported during epidemiological weeks 1–52 of 2024, of which 64 were confirmed [8].

The classical manifestations of mpox include fever, chills, headache, lymphadenopathy, myalgia, and a painful cutaneous eruption that progresses from macules to papules, vesicles, pustules, umbilicated pustules, and eventually crusts [9]. However, significant variation was observed during the 2022 outbreak, and the MPV has spread to diverse geographic regions, including tropical areas of Latin America, Africa, and Southeast Asia [10]. The differential diagnosis of mpox can be challenging across different geographic settings, as its clinical manifestations may overlap with those of several other infectious diseases [9].

We present nine cases of patients initially evaluated for suspected mpox. Following negative MPV PCR results, alternative diagnoses were established based on clinical and laboratory findings.

## 2. Materials and Methods

### 2.1. Ethics Statement

This study was conducted in accordance with the Declaration of Helsinki and approved by the Fundação Oswaldo Cruz (FIOCRUZ Minas) Research Ethics Committee (protocol no. 61832222.1.0000.5091 on 23 November 2022). Written informed consent was obtained from all participants.

### 2.2. Setting

Patients with suspected mpox were referred from public healthcare facilities throughout Minas Gerais to Eduardo de Menezes Hospital (HEM), the state’s referral center for infectious diseases, and were subsequently enrolled in the study. All participants were admitted to the infectious disease ward except patient G. The duration of illness prior to admission ranged from 4 to 21 days.

### 2.3. Procedure

We reviewed the medical charts of 30 patients with suspected mpox between July 2022 and October 2025. All patients underwent collection of fragments from lesions and MPV quantitative polymerase chain reaction (qPCR) testing, and this series includes those in whom MPV infection was excluded and an alternative diagnosis was established. The series includes nine patients (four women and five men), aged 19–58 years. The cases were identified within an ongoing prospective study of patients with suspected mpox, for which all participants provided written informed consent.

Samples were analyzed at a public health laboratory within the Brazilian Unified Health System (Sistema Único de Saúde, Belo Horizonte, Minas Gerais, Brazil [SUS]). MPV infection was ruled out using qPCR with the 5PLEX OPXV/MPV/VZV/MOCV/RP kit Bio-Manguinhos (Instituto de Tecnologia em Imunobiológicos, Fundação Oswaldo Cruz, Rio de Janeiro, Brazil). This multiplex assay enables differential detection of orthopoxviruses (OPVs), MPV, varicella-zoster (VZV), and molluscum contagiosum (MOCV); RNase P (RP) is included as an endogenous internal control [9]. The assay targets the OPG083 gene (I7L protein) for generic OPV detection and the OPG 68 gene (IMV membrane protein E6) [11] for MPV-specific detection. For arbovirus detection, the IBMP Biomol ZDC One kit, from Curitiba, Brazil, was used; this is a multiplex reverse transcription quantitative polymerase chain reaction (RT-qPCR) for Zika, Dengue, and Chikungunya viruses. Chikungunya serology was performed using the Anti-CHIKV ELISA IgM from Euroimmun, Lübeck, Germany. Syphilis testing was conducted using the Sífilis Assure Test (Assure Tech, Hangzhou, China) and VDRL (Wama Diagnóstico, São Paulo, Brazil). Testing for hepatitis B was performed with the HBsAg Assure test (Assure Tech,), while hepatitis C testing used the HCV Global X kit (Global X, São Paulo, Brazil), and human immunodeficiency virus (HIV) testing was conducted using the HIV 1+O Assure test (Assure Tech,), HIV 1.2 Global X (Global X), and the Imunoblot DPP Rapid HIV 1/2 kit from Bio-Manguinhos. When performed, biopsy samples underwent histopathological examination by AFIP Medicina Diagnostica, Brazil. Serological screening for anti-human T-lymphotropic virus (HTLV)-1/2 antibodies was performed by chemiluminescent immunoassay (CLIA) at Hermes Pardini Laboratory, a College of American Pathologists (CAP)-accredited national reference laboratory, Belo Horizonte, Brazil.

## 3. Results

Of the 30 patients with suspected mpox, nine were included in this case series because MPV PCR testing was negative and an alternative diagnosis was established. None of the cases reported here presented with lymphadenopathy or a history of upper respiratory tract infection. All patients were tested for mpox by PCR, and tested for HIV, hepatitis, HTLV, and syphilis. All patients except patient G were hospitalized.

Patient A. A 32-year-old man who has sex with men (MSM) with AIDS presented with disseminated skin lesions after discontinuing antiretroviral therapy 20 days earlier. The patient reported progressive worsening of the cutaneous lesions over the previous two weeks, accompanied by otitis with purulent discharge. Dermatological examination revealed erythematous scaly plaques involving the axillae, groin, buttocks, lower extremities, palms, and dorsal aspects of the hands, with scaling of the nasolabial folds and ears. Coral-red fluorescence under Wood’s lamp examination was observed in the axillae, groin, and buttocks. A draining sinus tract with purulent discharge was present in the left axilla, along with erythema and edema of the glans penis. Scattered papules and pustules were noted on the trunk, predominantly involving the lower extremities (Figure 1A) and groin. The histological examination of skin biopsy revealed hyperkeratosis, irregular acanthosis, mild dermal fibrosis, and a superficial perivascular lympho-histiocytic inflammatory infiltrate, supporting the diagnosis of chronic inflammatory dermatosis. Following discussion with the hospital’s dermatology team, we confirmed that the patient had previously been diagnosed with psoriasis and was already under dermatological follow-up for this condition. However, this previous diagnosis was not recognized at the time of hospital admission, when the patient was evaluated for suspected mpox. Inverse psoriasis is a chronic inflammatory dermatosis affecting intertriginous areas, characterized by well-demarcated, smooth erythematous plaques with little or no scaling. Genital involvement may resemble infectious dermatoses [12].

Patient B. A 43-year-old woman reported arthralgia involving the wrists, knees, ankles, and feet beginning approximately 1.5 months before presentation. At the same time, erythematous lesions appeared beneath the breasts and subsequently spread to the face, anterior and posterior trunk, and flexural areas, with mild scaling and pruritus. She was diagnosed with syphilis and treated with benzathine penicillin. Three days after the third dose of benzathine penicillin, she developed hematuria and foul-smelling urine and was referred for further evaluation, when anemia was identified. Skin lesions remained at different stages of evolution, including erythematous scaly plaques involving the inframammary folds, neck, extensor surfaces of the elbows, retro-auricular regions, umbilicus, abdomen, back, and lateral thighs, plantar surfaces of the feet, and distal lower extremities (Figure 1B), along with diffuse erythematous papules on the face, some covered by honey-colored crusts. Laboratory tests made 15 days before presentation showed a positive chikungunya PCR, total bilirubin of 1.68 mg/dL (reference range [RR], 0.0–1.2), C-reactive protein (CRP) of 192.6 mg/L (RR, <5), hemoglobin of 6.7 g/dL (RR, 12–15), leukocyte count of 21,580/mm^3^ (RR, 4000–10,000), and platelet count of 682,000/mm^3^ (RR, 150,000–400,000). On admission, laboratory tests revealed a non-reactive HIV test, reactive syphilis serology, albumin of 2.2 g/dL (RR, 1.0–2.5), alkaline phosphatase of 232 U/L (RR, 30–120), gamma-glutamyl transferase (GGT) of 117 U/L (RR, 5–50), CRP of 175 mg/L (RR, <5), hemoglobin of 5.7 g/dL (RR, 12–15), hematocrit of 19.2% (RR, 36–47), leukocyte count of 10,500/mm^3^ (RR, 4000–10,000), and platelet count of 664,000/mm^3^ (RR, 150,000–400,000). The patient was referred to as outpatient follow-up with the dermatology clinic and was clinically diagnosed with psoriasis vulgaris and inverse psoriasis, complicated by pustular psoriasis secondary to the use of systemic corticosteroid therapy and superimposed seborrheic dermatitis. Systemic corticosteroid therapy had been initiated by the infectious diseases team in the context of post-chikungunya arthralgia. The skin lesions gradually improved during follow-up with methotrexate therapy.

Patient C. A 35-year-old woman presented with multiple tender, erythematous–edematous papules and plaques diffusely distributed on her body. The lesions were raised and painful upon palpation, but the mucous membranes were spared. The tender, erythematous–edematous papules and plaques were prominent throughout the skin, predominantly affecting the upper and lower extremities (Figure 1C). Annular lesions were observed on the face. Although the patient remained afebrile throughout the clinical evaluation, a clinical diagnosis of acute febrile neutrophilic dermatosis, commonly known as Sweet’s syndrome, was established [13,14]. Laboratory findings revealed a leukocyte count of 11,500 cells/µL (RR, 4000–10,000), CRP of 271 mg/L (RR, <5), aspartate aminotransferase (AST) of 93 U/L (RR, 5–30), and alanine aminotransferase (ALT) of 125 U/L (RR, 5–35). The patient was treated with prednisone at 1 mg/kg/day (50 mg/day), followed by tapering to 40 mg/day, with a good clinical response. Ten days after treatment initiation, the erythema and pain had markedly improved, and the patient reported no residual symptoms.

Patient D. A 58-year-old man presented with acute onset of myalgia, fever, asthenia, and arthralgia, accompanied by multiple papules and crusted lesions on exposed areas, predominantly affecting the feet (Figure 1D). The lesions were painless and pruritic and were initially considered compatible with possible arthropod bites. Physical examination revealed edema of the left foot and petechiae on both lower limbs that did not blanch upon pressure. The patient presented clinical findings consistent with cellulitis of the foot and was treated with intravenous ceftriaxone (1 g every 12 h). Marked clinical improvement was observed by the third day of antibiotic therapy, with a substantial reduction in local inflammation and edema. Serum protein analysis showed mildly reduced total protein (5.7 g/dL, RR, 6.0–8.0) and albumin levels (3.0 g/dL, RR, 3.5–5.5), with an albumin/globulin (A/G) ratio of 1.1 (RR, 1.0–2.5). Laboratory evaluation demonstrated marked systemic inflammation, including CRP (205.5 mg/L, RR, <5) and procalcitonin (295.0 ng/mL, RR, ≤0.10), associated with leukocytosis (12,700/µL, RR 4000–10,000) and increased immature neutrophils. Liver biochemistry revealed mild elevations of AST (51 U/L, RR, 5–30) and ALT (46 U/L, RR, 5–35), with marked increases in GGT (396 U/L, RR, 5–35), alkaline phosphatase (165 U/L, RR, 30–120), and lipase (287 U/L, RR, 10–60). Serological testing confirmed Chikungunya virus infection.

Patient E. A 44-year-old woman was referred from the outpatient clinic after returning from a trip to Natal, Brazil, a coastal city. Ten days before hospital admission, she developed erythema, edema, pain, and pruritus involving the fifth toe. Initial treatment included prednisone and topical fusidic acid. Subsequently, the lesions progressed, with erythema, edema, pain, and pruritus extending to the dorsum of the foot, where serpiginous, thread-like tracks became evident (Figure 1E). Because of the inflammatory appearance of the foot lesions, together with two small, crusted lesions on the epigastric region, mpox was initially considered in the differential diagnosis. Initial laboratory evaluation demonstrated leukocytosis (13,600/µL, RR, 4000–10,000) with neutrophilia (78%), whereas renal and liver function tests, hemoglobin, and platelet count were within normal limits. Further clinical assessment, including the characteristic serpiginous morphology of the foot lesions, the history of recent exposure to sandy beaches, and the presence of similar lesions in her husband, supported the diagnosis of cutaneous larva migrans [15]. The patient was treated with ivermectin, followed by cephalexin because of suspected secondary bacterial infection, resulting in partial improvement of pain and pruritus.

Patient F. A 19-year-old man was admitted with myalgia and asthenia, with disseminated skin lesions affecting the mouth and genital region. Given the mucocutaneous involvement, he was hospitalized and placed under isolation precautions because of the initial suspicion of mpox. Multiple bullous lesions on an erythematous base, some ulcerated and crusted, involved the oral mucosa. Lesions on an erythematous base were present on the palms (Figure 1F) and soles. There were also ulcerated erythematous lesions involving the glans penis, penile shaft, and scrotum, without drainage or exudate. He was treated with topical corticosteroids for five days, acyclovir, and oral amoxicillin–clavulanate. The diagnosis of erythema multiforme was established based on the characteristic clinical appearance of the lesions [16].

Patient G. A 44-year-old man who has sex with men and has well-controlled HIV infection was diagnosed with mpox one year prior to his current syphilis diagnosis. Diffuse erythematous lesions affecting the palms and soles (Figure 1G) raised suspicion of a new episode of mpox. A reactive Sifilis Assure Test and a VDRL titer of 1:64 confirmed the diagnosis of syphilis.

Patient H. A 32-year-old woman presented with community-acquired pneumonia; chest radiography revealed bilateral infiltrates involving the lower thirds of both lungs. Laboratory evaluation revealed mild anemia (hemoglobin 11.5 g/dL, RR, 12–15, hematocrit 34.7%, RR, 36–47), markedly elevated CRP (115 mg/L, RR, <5), and respiratory alkalosis on arterial blood gas analysis (pH 7.49, RR, 7.35–7.45; PaCO_2_ 24 mmHg, RR, 35–45; HCO_3_^−^ 18.3 mmol/L, RR, 22–27). HIV serology was reactive by both the anti-HIV-1/2 antibody assay and the DPP HIV-1/2 rapid immunoblot, with gp160 and gp41 bands detected. On the day following hospital admission, high-dose trimethoprim–sulfamethoxazole was started for suspected Pneumocystis jirovecii pneumonia. On the third hospital day, the patient required endotracheal intubation and invasive mechanical ventilation because of worsening respiratory failure. One week before hospital admission, the patient developed skin lesions. Dermatological examination revealed multiple disseminated skin lesions characterized by violaceous centers surrounded by erythematous borders, raising the possibility of erythema multiforme. The lesions were predominantly located on the medial aspect of the right thigh, the dorsum, and the upper lip (Figure 1H). Given their clinical appearance, mpox was also considered in the differential diagnosis because of the umbilicated morphology of some lesions. Swabs were collected from skin lesions, and the PCR result was positive for varicella-zoster virus (VZV). Four days after admission, intravenous acyclovir was initiated at a dose of 10 mg/kg every 8 h.

Patient I. A previously healthy 26-year-old man, not previously immunized against varicella, presented with a 3-day history of fever, myalgia, and headache. He also reported a 7-day history of pleomorphic skin lesions that initially appeared as vesicles on the thighs and subsequently spread to the upper limbs, trunk, and face (Figure 1I), evolving into pustules and crusts. A swab was collected from one of the lesions, and PCR testing was positive for varicella-zoster virus (VZV), confirming the diagnosis of varicella.

To illustrate the clinical presentation of confirmed mpox for comparison with the cases described here, we present data from patient G’s previous mpox episode, which occurred about a year before the episode reported in this case series. At that time, patient G was diagnosed with mpox and reported a three-day prodrome characterized by fever, followed by the development of a vesiculopapular rash initially involving the head and hands, with subsequent extension to the trunk and other areas of the body. Oral lesions on the following day were identified, leading to dysphagia. The patient reports pain, itching, fever, headache, myalgia, and malaise. The lesions were firm, deep-seated, well-circumscribed, painful, pruritic, and sometimes umbilicated (Figure 2). He denied sexual contact and did not identify anyone with mpox among his contacts. The patient was in home isolation, and ART was continued (tenofovir/lamivudine and dolutegravir). MPV infection was confirmed by qPCR using a swab of vesicular fluid. After 9 days, the patient reported plantar lesions with ambulation problems due to pain and had more than 30 lesions distributed throughout the body. Lymphadenopathy was not prominent.

The lesions showed different stages of evolution and no signs of secondary infection. Crusts were present on the trunk and face, with vesicles on the hands, palms, and soles. Fever resolved on the 9th day of symptoms. Laboratory tests showed a normal white blood cell count (6680/mm^3^) although there was mild neutropenia (1760/mm^3^). All other biochemical parameters were within normal limits, including glucose, urea, creatinine, sodium, potassium, and AST/ALT. The CD4 count was 669/μL, and the plasma HIV-1 viral load was 30 copies/mL. Serologic tests for syphilis and hepatitis C were both negative (Table 1). The clinical course was favorable despite coinfection with HIV, as the patient had a good immunological status. In Table 1, we present a comparison of the clinical features of Patient G during the laboratory-confirmed mpox episode and the subsequent syphilis episode.

## 4. Discussion

During the 2022 global mpox outbreak, heightened clinical awareness resulted in increased suspicion of mpox among patients presenting with vesicular, pustular, ulcerative, or crusted skin lesions. However, the clinical spectrum of mpox is broad and influenced by several factors, including the stage of infection, immune status, vaccination history, and route of transmission. Patients may present with localized or atypical lesions, limited systemic symptoms, or isolated genital, perianal, or extragenital involvement [17], which can closely resemble sexually transmitted infections, bacterial skin infections, viral diseases, and inflammatory dermatoses. Therefore, distinguishing mpox from other conditions with similar clinical manifestations remains challenging, particularly in tropical and endemic regions where multiple infectious and inflammatory diseases coexist [18]. The present case series highlights the diversity of conditions initially suspected as mpox in a tropical referral center. Although all patients presented clinical features suggestive of mpox, laboratory investigations combined with thorough infectious disease and dermatologic assessment established alternative diagnoses, highlighting the importance of a systematic differential diagnostic approach. Patient G further illustrates this diagnostic challenge, as a subsequent episode of syphilis clinically mimicked his previous laboratory-confirmed mpox infection, emphasizing the role of etiological testing in distinguishing clinically overlapping conditions. Clinical assessment remains essential; however, molecular testing for MPV should be performed to confirm infection. Integration of epidemiological information, lesion morphology, laboratory findings, and, when indicated, histopathological evaluation can improve diagnostic accuracy and guide appropriate patient management.

The differential diagnosis of mpox includes several infectious conditions, particularly those associated with vesicular, pustular, ulcerative, or crusted lesions. Commonly recognized mimickers include varicella, herpes simplex virus infection, syphilis, and acute retroviral syndrome [19]. Other infections, including measles, scabies, impetigo, and ecthyma, should also be considered according to the clinical presentation and epidemiological setting [6]. In our series, ectoparasitic diseases represented relevant diagnostic challenges, as demonstrated by the patient with cutaneous larva migrans [15]. This condition may present with pruritic inflammatory lesions and be mistaken for mpox, particularly in tropical environments [20].

Beyond infectious diseases, inflammatory dermatoses may occasionally mimic mpox and should be considered when evaluating patients with atypical eruptions. Although psoriasis is not among the most frequently reported differential diagnoses of mpox, extensive or atypical psoriatic lesions may lead to clinical suspicion of mpox and unnecessary isolation or antiviral investigation [21]. Psoriasis [12] was among the inflammatory conditions identified in our case series, highlighting the importance of considering chronic inflammatory dermatoses during outbreak settings. Similarly, erythema multiforme should be included in the differential diagnosis, particularly in patients presenting with acute polymorphic mucocutaneous lesions that may overlap clinically with mpox-associated eruptions [22]. Misclassification of suspected mpox cases may result in unnecessary isolation measures, delayed recognition of alternative diagnoses, inappropriate management, and increased healthcare costs.

This study has some limitations. It is a small, single-center case series from a referral hospital, which may limit the generalizability of the findings. In addition, its descriptive design does not allow a systematic comparison between these cases and patients with confirmed mpox. Nevertheless, the series reflects real-world diagnostic challenges among patients referred to suspected mpox.

## 5. Conclusions

This case series demonstrates that various infectious and non-infectious conditions can resemble mpox, making clinical differentiation challenging. Features such as lymphadenopathy, characteristic lesion morphology, and anogenital involvement may raise suspicion for mpox but are nonspecific and can overlap with other diseases. Additionally, some patients may present with more than one condition, further complicating the diagnosis. Timely laboratory confirmation is essential for appropriate clinical management, as it can help reduce unnecessary isolation and potentially avoidable hospital admissions.

## Figures and Tables

**Figure 1:**
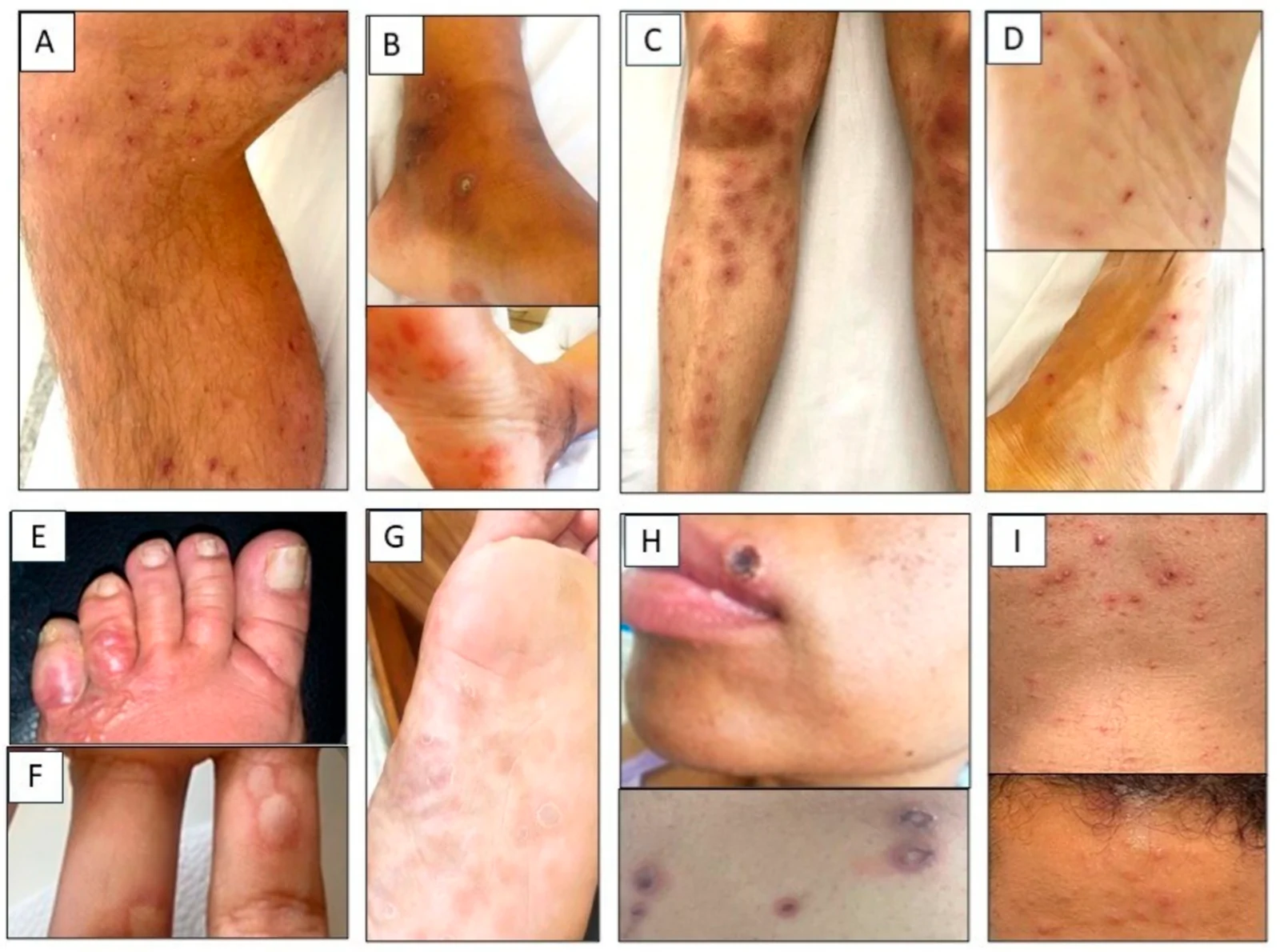
Lesions initially suspected of being mpox (all with negative PCR results from lesion samples) and their respective diagnoses. (**A**) Inverse psoriasis; (**B**) Syphilis, Chikungunya, psoriasis vulgaris and inverse psoriasis; (**C**) Sweet’s syndrome; (**D**) Chikungunya; (**E**) Larva migrans; (**F**) Erythema multiforme; (**G**) Syphilis. PCR-confirmed VZV infection in patient (**H**) (herpes zoster) and patient (**I**) (varicella).

**Figure 2:**
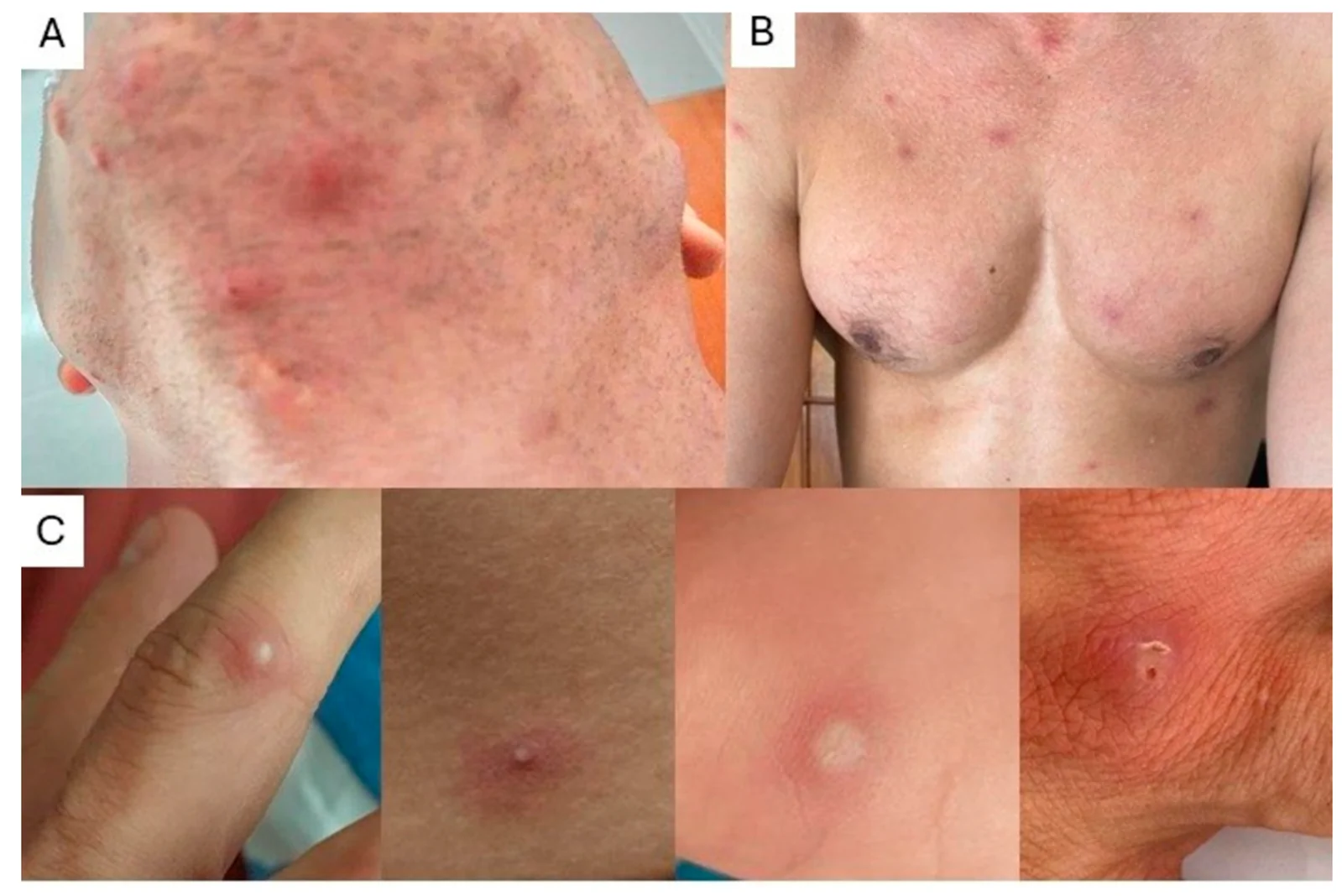
Evolution of mpox lesions in Patient G during the mpox episode. Mpox lesions on: (**A**) anterior cervical region; (**B**) chest; (**C**) at different stages.

**Table 1:** Comparison of the laboratory-confirmed mpox episode and the subsequent mpox-mimicking syphilis episode in Patient G.

Clinical Characteristic	Mpox	Mpox-Mimicking (Syphilis)
Final diagnosis	Mpox	Syphilis
Prodrome	3 days of fever before rash	No
Fever	Yes; remission on day 9 of symptoms	No
Headache	Yes	No
Myalgia	Yes	No
Malaise	Yes	No
Pain	Yes	No
Pruritus	Yes	No
Initial lesion distribution	Head and hands, subsequently extending to the body	Diffuse erythematous lesions affecting palms and soles
Oral lesions	Yes; followed by dysphagia	No
Palmar involvement	Yes	Yes
Plantar involvement	Yes; painful lesions causing difficulty walking	Yes
Lesion morphology	Vesiculopapular; firm, deep-seated, well-circumscribed; sometimes umbilicated; vesicles and crusts	Diffuse erythematous lesions
Lesions in different evolutionary stages	Yes	No
Lesion burden	>30 lesions distributed throughout the body	Yes
Lymphadenopathy	Not prominent	Absent
Signs of secondary infection	No	No
Syphilis testing *	Negative	Positive
MPV qPCR	Positive	Negative
Clinical outcome	Favorable	Favorable

* The treponemal test was positive, and the VDRL titer was 1/64.

## Data Availability

The data that support the findings of this study are available on request from the corresponding author [SHC]. Data are not publicly available since such information could compromise the privacy of the patients.

## Abbreviations

CAP	College of American Pathologists
CLIA	chemiluminescent immunoassay
CRP	C-reactive protein
HBsAg	hepatitis B surface antigen
HCV	hepatitis C virus
HEM	Hospital Eduardo de Menezes
HIV	human immunodeficiency virus
HTLV	human T-lymphotropic virus
ICTV	International Committee on Taxonomy of Viruses
IMV	intracellular mature virion
LDH	lactate dehydrogenase
MPV	monkeypox virus
MSM	men who have sex with men
OPV	orthopoxvirus
PCR	polymerase chain reaction
PHEIC	Public Health Emergency of International Concern
qPCR	quantitative polymerase chain reaction
RR	reference range
RT-qPCR	reverse transcription quantitative polymerase chain reaction
SUS	Brazilian Unified Health System (Sistema Único de Saúde)
VDRL	Venereal Disease Research Laboratory
VZV	varicella-zoster virus
WHO	World Health Organization
